# ﻿Additions to the saprobic fungi (Ascomycota) associated with macadamia trees from the Greater Mekong Subregion

**DOI:** 10.3897/mycokeys.113.140031

**Published:** 2025-01-24

**Authors:** Xian Zhang, Saowaluck Tibpromma, Samantha C. Karunarathna, Tian-Ye Du, Li-Su Han, Abdallah M. Elgorban, Jaturong Kumla, Chanokned Senwanna, Dong-Qin Dai, Nakarin Suwannarach, Hao-Han Wang

**Affiliations:** 1 Key Laboratory of Yunnan Provincial Department of Education of the Deep-Time Evolution on Biodiversity from the Origin of the Pearl River, College of Biology and Food Engineering, Qujing Normal University, Qujing 655011, China; 2 Department of Biology, Faculty of Science, Chiang Mai University, Chiang Mai 50200, Thailand; 3 Center of Excellence in Microbial Diversity and Sustainable Utilization, Chiang Mai University, Chiang Mai 50200, Thailand; 4 Center of Excellence in Fungal Research, Mae Fah Luang University, Chiang Rai 57100, Thailand; 5 Department of Botany and Microbiology, College of Science, King Saud University, P O Box 2455, Riyadh, 11451, Saudi Arabia; 6 Office of Research Administration, Chiang Mai University, Chiang Mai 50200, Thailand

**Keywords:** Dothideomycetes, morphology, multigene phylogeny, new taxa, saprobes, Sordario­mycetes, taxonomy

## Abstract

Macadamia trees, the most economically important Proteaceae perennial crop, are globally renowned for their edible nuts. During our surveys of microfungi associated with macadamia in China and Thailand, we isolated three saprobic fungi from dead macadamia branches. Our multigene phylogenetic analyses (ITS, LSU, SSU, *tef*1-α, *TUB*2, and *ACT* loci), genealogical concordance phylogenetic species recognition (GCPSR) with a pairwise homoplasy index (PHI) test, and morphological characteristics led to the discovery of two new species, *Dothiorellamacadamiae* and *Phaeoacremoniumchiangmaiense*, and one new record, *Melomastiapuerensis*. We provide morphological descriptions, photo plates, phylogenetic analysis results, and PHI test results of the two new species, along with comparisons with closely related taxa. These findings have global implications for understanding the diversity of microfungi associated with macadamia trees.

## ﻿Introduction

*Macadamia* F. Muell. is one of the perennial crops that produces an edible nut, which is native to Australia and later widely distributed in frost-free tropical and subtropical regions (Wrona et al. 2020; Li et al. 2022). The genus is composed of four species, i.e., *Macadamiaintegrifolia* Maiden & Betch, *M.jansenii* C.L.Gross & P.H.Weston, *M.ternifolia* F. Muell., and *M.tetraphylla* L.A.S. Johnson (Mast et al. 2008; Prasannath et al. 2020; Prasannath et al. 2021; Tao et al. 2022). *Macadamiaintegrifolia* and *M.tetraphylla* are well known and can produce edible nuts, while *M.jansenii* and *M.ternifolia* produce small inedible kernels (Prasannath et al. 2021). It is crucial to note that *M.jansenii* is an endangered and poisonous tree, and *M.ternifolia* was listed as a vulnerable tree (Costello et al. 2009), underscoring the importance of our collective responsibility to preserve these species.

*Macadamia* was initially introduced from Australia and trial-planted in China in the 1970s (Ma et al. 2021; Shuai et al. 2022). By the end of 2018, China’s macadamia plantation area was more than 301,206 km^2^, accounting for more than one-third of the world’s planting area (Ma et al. 2021; Tao et al. 2022). China is the world’s largest and fastest-growing macadamia-cultivating country (Tao et al. 2022). The most-grown *Macadamia* species in southern China is *M.integrifolia*, distributed in the Guangxi and Yunnan provinces (Hong et al. 2018; Zhong et al. 2020; Tao et al. 2022). In Thailand, the first attempt to grow macadamia was initiated in Chiang Mai Province in 1953, but it failed (Supamatee et al. 1992; Hardner et al. 2009; https://www.de-loei.com/history-of-macadamia-nuts). In 1981, macadamia nuts were officially introduced to Thailand, and in 1989, Thailand began large-scale cultivation of Australian nuts (over 1,000 trees) and established internationally standardized processing plants. That same year, the Doi Tung development project in Mae Fah Luang District, Chiang Rai Province, was the first project to be implemented (https://www.de-loei.com/history-of-macadamia-nuts).

Microfungi are one of the key organisms in forest ecosystems, and they are distributed worldwide with a very high diversity (Bahram and Netherway 2022). In recent years, several microfungi have been reported from macadamia, including different life modes such as endophytic, pathogenic, and saprobic; most of them are mainly focused on macadamia-associated pathogenic fungi, while saprobic and endophytic fungi have been poorly studied (Prasannath et al. 2020, 2021; Li et al. 2022, 2023; Zhang et al. 2024). Only a few species have been introduced based on the USDA Fungal Database, with a comprehensive collection of fungal species and their characteristics (https://fungi.ars.usda.gov/), viz. *Fusariumpolyphialidicum* Marasas, P.E. Nelson, Toussoun & P.S. van Wyk and *Neopalmiascomamacadamiae* X. Zhang, K.D. Hyde, Tibpromma & Karunarathna (Ruiz et al. 1997; Zhang et al. 2024).

In this study, we aim to introduce two new species and one new host record of saprobic fungi found on dead twigs of *Macadamia* spp. based on morphological characteristics, multi-locus phylogeny analyses of the combined ITS, LSU, SSU, *tef*1-α, *TUB*2, and *ACT* sequence data, and genealogical concordance phylogenetic species recognition (GCPSR) with a pairwise homoplasy index (PHI).

## ﻿Materials and methods

### ﻿Specimen collection and morphological study

Dead twigs of *Macadamia* spp. with fungal fruiting bodies were collected from the Yunnan Province of China and the Chiang Mai Province of Thailand. Each sample was placed in a separate plastic bag, marked with information such as collection site, collection date, collection altitude, and global positioning system (GPS) (Rathnayaka et al. 2024), and taken to the mycology laboratory at Qujing Normal University and Chiang Mai University. The fruiting body structures were observed by a LEICA S8 APO optical microscope (Olympus, Tokyo, Japan). Micro-morphological characteristics were observed by a compound microscope (OLYMPUS BX53, Olympus, Tokyo, Japan), and photographs were taken from the OLYMPUS DP74 (Olympus, Tokyo, Japan) digital camera fitted to the compound microscope. The microstructures were measured using Tarosoft (R) Image Frame Work (v.0.9.0.7) software, and the photo plates were made in Adobe Photoshop CS3 Extended version 10.0 software (Adobe Systems, USA). Single spore isolation was carried out following the method described by Senanayake et al. (2020), and the pure culture was obtained from potato dextrose agar (PDA) after one month. Herbarium specimens were deposited at the Herbarium of Guizhou Medical University (GMB) and in the Herbarium of the Department of Biology (CMUB), Faculty of Science, Chiang Mai University. Living cultures were deposited at the Sustainable Development of Biological Resources (SDBR-CMU), Faculty of Science, Chiang Mai University, Thailand. The Index Fungorum numbers (IF) and Facesoffungi (FoF) numbers were obtained as per the instructions provided in Index Fungorum (2025) and Jayasiri et al. (2015), respectively.

### ﻿DNA extraction, PCR amplification, and sequencing

DNA was extracted from the three-week-old pure cultures growing on PDA, or fungal fruiting bodies were used when the pure cultures could not be obtained. Genomic DNA extraction was obtained using the Biospin Fungus Genomic DNA Extraction Kit-BSC14M1 (BioFlux®, P.R. China), following the manufacturer’s guidelines and preserved at -20 °C for long-term use. The different gene regions, primers, and PCR thermal cycle programs for amplification are detailed in Table 1. The PCR amplification in China has a total volume of 25 μL, including ddH_2_O (9.5 μL), 2× Master Mix (12.5 μL) (Bioteke Corporation, Beijing, China), DNA template (1 μL), and each reverse and forward primer (1 μL) (Tibpromma et al. 2018), and while in Thailand, the total volume is 20 μL, including ddH_2_O (6 μL), 2× Quick Taq^TM^ HS DyeMix (10 μL) (TOYOBO, Japan), DNA template (2 μL), and each reverse and forward primer (1 μL) (Senwanna et al. 2023). PCR product sequencing and purification were performed at Sangon Biotech (Shanghai, Co., Ltd.), China, and 1^st^ BASE Company (Kembangan, Malaysia), respectively.

**Table 1.** Genes, primers, and PCR conditions in this study.

Genes	Primers	PCR conditions	References
ITS	ITS5/ITS4	95 °C: 3 mins, (94 °C: 30 s, 55 °C: 50 s, 72 °C: 90 s) × 35 cycles, 72 °C: 10 mins (*Phaeoacremonium*)	White et al. (1990)
		95 °C: 2 mins, (95 °C: 30 s, 52 °C: 30 s, 72 °C: 60 s) × 35 cycles, 72 °C: 10 mins (*Dothiorella*)	
LSU	LR0R/LR5	95 °C: 3 mins, (94 °C: 30 s, 55 °C: 50 s, 72 °C: 90 s) × 35 cycles, 72 °C: 10 mins (*Phaeoacremonium*, *Melomastia*)	Vilgalys and Hester (1990)
SSU	NS1/NS4	95 °C: 3 mins, (94 °C: 30 s, 55 °C: 50 s, 72 °C: 90 s) × 35 cycles, 72 °C: 10 mins (*Melomastia*)	White et al. (1990)
*tef*1-α	983F/2218R	95 °C: 3 mins, (95 °C: 30 s, 55 °C: 50 s, 72 °C: 90 s) × 35 cycles, 72 °C: 10 mins (*Phaeoacremonium*, *Melomastia*)	Rehner and Buckley (2005)
	728F/986R	95 °C: 3 mins, (95 °C: 30 s, 55 °C: 30 s, 72 °C: 60 s) × 40 cycles, 72 °C: 10 mins (*Dothiorella*)	Carbone and Kohn (1999)
*TUB*2	T1/Bt2b	94 °C: 4 mins, (94 °C: 40 s, 52 °C: 30 s, 72 °C: 60 s) × 35 cycles, 72 °C: 10 mins (*Phaeoacremonium*, *Dothiorella*)	Glass and Donaldson (1995); O’Donnell and Cigelnik (1997)
*ACT*	ACT-512F/ACT-783R	94 °C: 4 mins, (94 °C: 40 s, 52 °C: 30 s, 72 °C: 60 s) × 35 cycles, 72 °C: 10 mins (*Phaeoacremonium*)	Carbone and Kohn (1999)

### ﻿Phylogenetic analyses

Sequences were assembled by the Geneious program (9.1.2) (https://www.geneious.com/), and the newly generated assembled sequences were copied to BLASTn for searches. The related sequences used in phylogenetic analyses were retrieved from GenBank following BLASTn search results and the latest publication (Xu et al. 2024). The sequence data alignments were aligned by the online multiple alignment program MAFFT version 7 (Katoh et al. 2019; https://mafft.cbrc.jp/alignment/server/) and improved manually wherever necessary in BioEdit v.7.0.5.2 (Hall 1999). The alignments were automatically adjusted using trimAl.v1.2rev59 (Capella-Gutiérrez et al. 2009). The Sequence Matrix program (1.7.8) was used to combine all sequence data (Vaidya et al. 2011). The ALignment Transformation EnviRonment (ALTER) was used to convert the FASTA format to PHYLIP and NEXUS format for maximum likelihood analyses (ML) and Bayesian inference analysis (BI), respectively (Larsson 2014).

The phylogenetic analyses of combined genes (ITS, *tef*1-α, and *TUB*2 for the *Dothiorella* dataset; ITS+*TUB*2+*ACT+tef*1-α+LSU for the *Phaeoacremonium* dataset; and LSU+SSU+*tef*1-α for the *Melomastia* dataset) were based on ML and BI analyses. The ML trees were performed using RAxML-HPC2 on XSEDE (8.2.12) (Stamatakis 2006, 2014; Stamatakis et al. 2008) with 1,000 rapid bootstrap replicates under the GTRGAMMA substitution model of evolution in the online CIPRES Science Gateway platform (https://www.phylo.org/portal2/login!input.action, Miller et al. 2010). The BI analyses were performed using MrBayes on XSEDE (8.2.12) (Stamatakis 2014), with the selection of best-fit models of evolution estimated by MrModelTest 2.2 (Nylander 2004) and PAUP v. 4.0b10 (Ronquist and Huelsenbeck 2003). Six simultaneous Markov chains were run for 2,000,000 generations, and trees were sampled for every 200^th^ generation. The run was automatically terminated when the standard deviation of split frequencies fell below 0.01. The initial 20% of the generated trees, representing the burn-in phase of the analysis, were excluded, and the other 80% of trees were used to calculate posterior probabilities in the majority rule consensus tree (Cai et al. 2006). The phylogenetic trees were visualized using FigTree v.1.4.0 (Rambaut 2012) and edited using Microsoft PowerPoint 2021 and Adobe Photoshop CS3 Extended version 10.0 software (Adobe Systems, USA). Maximum likelihood (ML) bootstrap values ≥ 60% and Bayesian posterior probabilities (PP) bootstrap values ≥ 0.90 are indicated above each branch.

### ﻿Pairwise homoplasy index test analysis

The pairwise homoplasy index (PHI) test using SplitsTree V4 (Huson and Bryant 2006) was used to assess the extent of recombination in newly identified *Dothiorella* species in comparison with closely related species (Bruen et al. 2006; Huson and Bryant 2006; Tian et al. 2021, 2024). A concatenated multi-locus dataset composed of closely related species was used for the analyses. Using the LogDet transformation and splits decomposition options, a phylogenetic network from the concatenated five datasets shows the relationships between closely related taxa. PHI results below 0.05 (Φw < 0.05) demonstrate significant recombination in the dataset (Hilário et al. 2021; Tian et al. 2024).

## ﻿Results

### ﻿Taxonomy and phylogenetic results

#### ﻿ Dothiorella

Taxon classificationFungiBotryosphaerialesBotryosphaeriaceae

Sacc.

22C86232-2F4F-573D-B3D0-6F104DBFAB89

##### Notes.

*Dothiorella* (Botryosphaeriaceae Theiss. & Syd.) was introduced by Saccardo (1880) with *D.pyrenophora* Berk. ex Sacc. as the type species. *Dothiorella* has 422 epithets listed in Index Fungorum (2024); however, only 59 species have available molecular data in GenBank. The members of *Dothiorella* can be found in a wide range of hosts, and taxa exist as endophytes, pathogens, and saprobes (Phillips et al. 2013; Dissanayake et al. 2016; Rathnayaka et al. 2022). The sexual morph of *Dothiorella* is characterized by ascospores that are hyaline to yellowish brown when immature and later become brown, and 1- or 2- septate and asexual morph is characterized by conidia that are initially hyaline and aseptate, later becoming brown and 1-septate, often attached to conidiogenous cells (Rathnayaka et al. 2022; Senanayake et al. 2023). In this study, we introduced one new species (*D.macadamiae*) in *Dothiorella*, which was isolated from the macadamia tree in Thailand. Additionally, our collection is the first report of *Dothiorella* species associated with macadamia.

#### ﻿ Dothiorella macadamiae

Taxon classificationFungiBotryosphaerialesBotryosphaeriaceae

X. Zhang & N. Suwannar. sp. nov.

E5895799-48FE-5931-9CC5-CA861302B00C

Index Fungorum: IF903216

Facesoffungi Number: FoF17086

Figs 3 , 4

##### Etymology.

“*macadamiae*” refers to the host plant genus *Macadamia*.

##### Holotype.

CMUB 40066.

##### Description.

***Saprobic*** on dead twigs of *Macadamia* sp. **Sexual morph: *Ascomata*** 80–120 × 160–220 µm (x̄ = 100 × 193 µm, *n* = 10), immersed, visible as dark dots on the host surface, under to clypeus, solitary, uni-loculate, ampulliform, papillate, without ostiole. ***Peridium*** 50–190 µm wide (x̄ = 95 µm, *n* = 25), comprising three section layers, the inner section layer composed of hyaline cells of ***textura angularis***, the outer section layer with brown to dark brown cells of ***textura angularis***, and the outermost layer of cells surrounding the ascomata is composed of brown cells of ***textura prismatica***. ***Hamathecium*** 4.5–8.5 µm wide (x̄ = 6.5 μm, *n* = 30), comprising cylindrical, hyaline, septate, cellular pseudoparaphyses. ***Asci*** 110–235 × 23–38 µm (x̄ = 173 × 32 µm, *n* = 30), 6–8-spored, bitunicate, clavate to broadly fusoid-ellipsoid, with furcated pedicel, apically rounded, with a well-developed ocular chamber. ***Ascospores*** (27–)30–37(–40) × 14–19 µm (x̄ = 33 × 17 µm, *n* = 55), overlapping, uniseriate, oval to ellipsoid, hyaline to yellowish brown, aseptate when young, becoming brown to dark brown, 1- or 2- septate at maturity, slightly constricted at the septum, smooth-walled, granular, with mucilaginous polar appendages at one or both ends. **Asexual morph: *Conidiomata*** pycnidial produced on PDA within seven weeks, solitary or aggregated, superficial, brown, hairy, globose to subglobose, covered with hyphal hairs, unilocular. ***Conidiophores*** reduce to conidiogenous cells. ***Conidiogenous cells*** holoblastic, discrete, cylindrical, hyaline, smooth-walled, proliferating percurrently. ***Conidia*** 19–26.5 × 10–13.5 µm (x̄ = 22.6 × 11.2 µm, *n* = 50), hyaline and aseptate when immature, brown to dark brown and one-septate when mature, oblong to ovoid, granular, one end obtuse to slightly rounded ends, one cell slightly wider or same width. ***Chlamydospores*** hyaline to brown, branched, with thickened, septate, brown to dark brown at the septa.

##### Culture characteristics.

Ascospores germinating on PDA within 24 h at 28 °C, colony on PDA reaching 9 cm diam. after two weeks at 28 °C, rough surface, hairy, cottony, and pale olivaceous grey from above, and grey to black in reverse.

##### Material examined.

Thailand • Chiang Mai Province, Doi Saket District, 18°52'43"N, 99°13'15"E, 384 m elevation, on a dead branch of *Macadamia* sp., 24 November 2023, Xian Zhang, TCMM25, CMUB 40066, holotype; ex-type living culture, SDBR-CMU512, other living culture SDBR-CMU513.

##### GenBank number.

SDBR-CMU512 = ITS: PQ699724, *tef*1-α: PQ758592, *TUB*2: PQ736693; SDBR-CMU513 = ITS: PQ699725, *tef*1-α: PQ758593, *TUB*2: PQ736694.

##### Notes.

**Figure 1.**
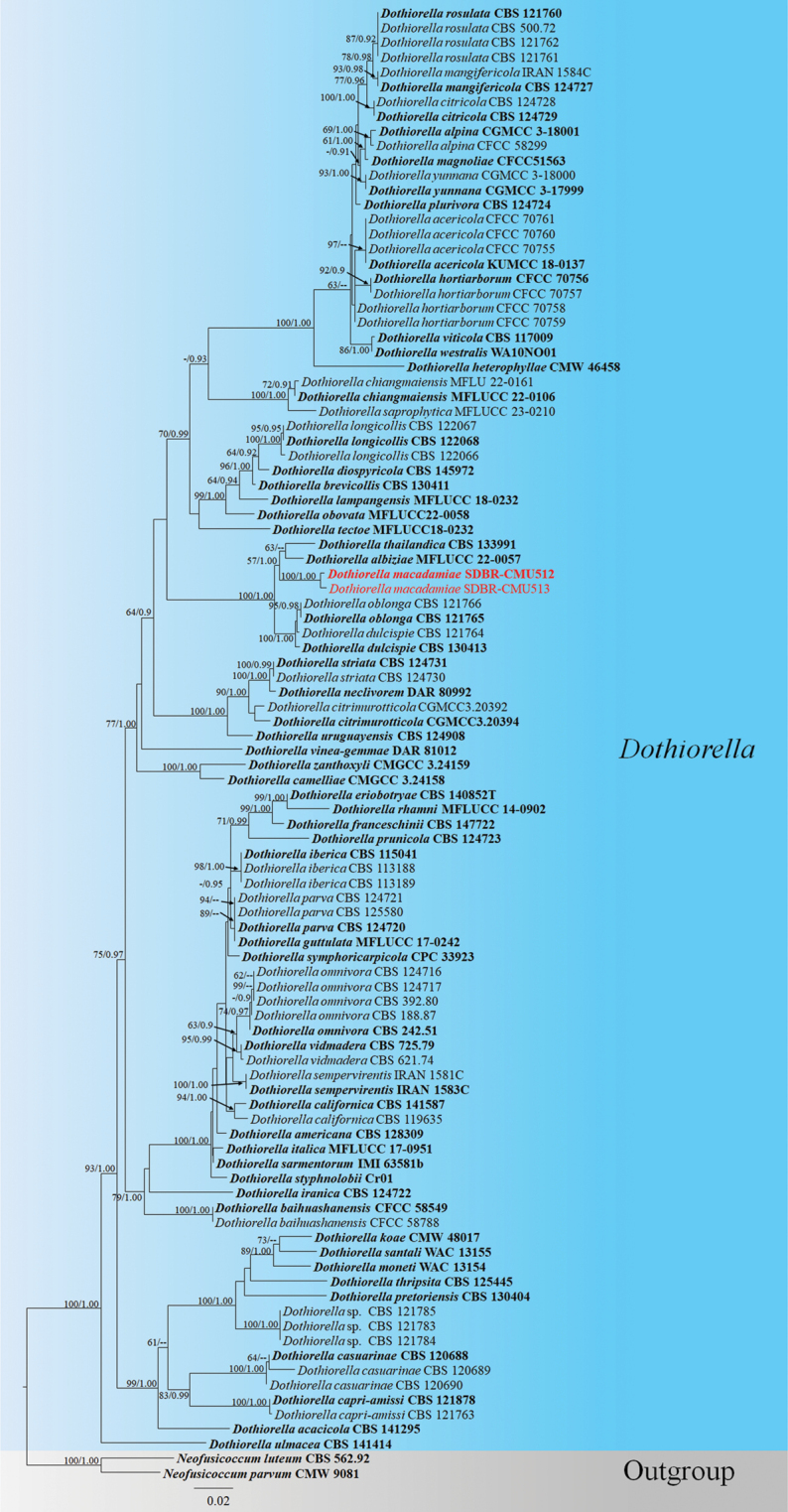
RAxML tree based on a combined dataset of ITS, *tef*1-α, and *TUB*2 gene sequences data, which comprised 1265 base pairs (ITS = 1–527 bp, *tef*1-α = 528–818 bp, *TUB*2 = 819–1265 bp). The best scoring RAxML tree with a final ML optimization likelihood value of -8702.022443 is presented. The matrix had 696 distinct alignment patterns, with 16.83% of undetermined characters or gaps. Estimated base frequencies were as follows: A = 0.209011, C = 0.306672, G = 0.250984, T = 0.233333; substitution rates: AC = 1.037189, AG = 2.254040, AT = 1.059283, CG = 1.045839, CT = 4.497195, GT = 1.000000; proportion of invariable sites I = 0.499857; gamma distribution shape parameter *α* = 0.610523. The ML analysis and Bayesian inference (BI) analyses showed nearly identical tree topologies, bootstrap support values for ML equal to or greater than 60%, and BI analysis values equal to or greater than 0.90 PP are given at each node. The tree is rooted with *Neofusicoccumluteum* (CBS 562.92) and *N.luteum* (CMW 41365). Newly generated species are shown in red, while the ex-type species are shown in bold. Remarks: The ML bootstrap value less than 60% is presented on the node of the new taxon.

**Figure 2.**
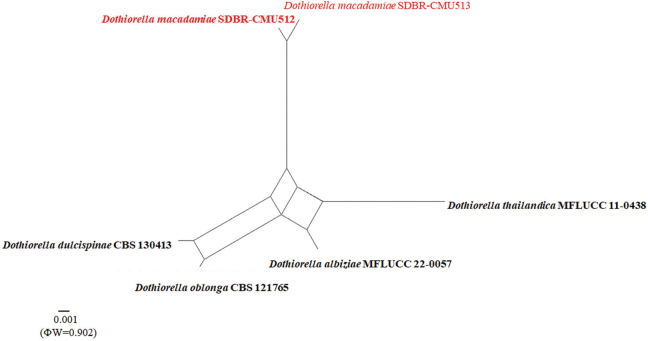
Results of the PHI test of *Dothiorellamacadamiae* and closely related species using both LogDet transformation and splits decomposition. The PHI test results (Φw) < 0.05 indicate significant recombination within the dataset. The new taxa are in red font, and bold indicates holotype or ex-type strains.

In the phylogenetic analyses, our isolate *D.macadamiae* forms an independent branch sister to *D.albiziae* and *D.thailandica* with 57% ML and 1.00 PP support (Fig. 1). Based on the BLASTn results of ITS sequences of our strain (SDBR-CMU512, ex-type), it is 99.64% similar to *D.oblonga* (CBS 121765); the *tef*1-α result is similar to *D.dulcispinae* (CMW:36462) with 90.11%, and the *TUB*2 result matched with *D.albiziae* (MFLU 22-0093) with 98.83% similarity. Our isolates of *D.macadamiae* formed an independent branch sister to *D.albiziae* and *D.thailandica* with the ML bootstrap support of 57% (Fig. 1). We carried out the PHI test to confirm the novelty of our new taxon and found no significant recombination event between our strain and the closely related taxa (Φw = 0.902) (Fig. 2). Also, our species (SDBR-CMU512) was compared in ITS, *tef*1-α, and *TUB*2 with *D.albiziae* (MFLUCC 22-0057) and *D.thailandica* (MFLUCC 11-0438) (Table 2) and found that the *tef*1-α gene shows more than 20 bp difference. Morphologically, *D.macadamiae* differs from *D.albiziae* by having a bigger (19–26.5 × 10–13.5 µm vs. 14–18 × 6–8 μm) and one cell slightly wider or the same width conidia; they share similar conidia, being oblong to ovoid (Rathnayaka et al. 2022). *Dothiorellamacadamiae* is distinguished from *D.thailandica* by having bigger (19–26.5 × 10–13.5 µm vs. 15–20 × 6.5–8 μm), granular, oblong to ovoid conidia but having similar hyaline conidiogenous cells (Liu et al. 2012). *Dothiorellaalbiziae* and *D.thailandica* have been recorded from their asexual morph. Therefore, we could not compare the sexual morphological characteristics of *D.macadamiae* with those of the two species. We introduce *D.macadamiae* as a new species based on morphology, nucleotide comparisons, and phylogenetic analyses.

**Figure 3.**
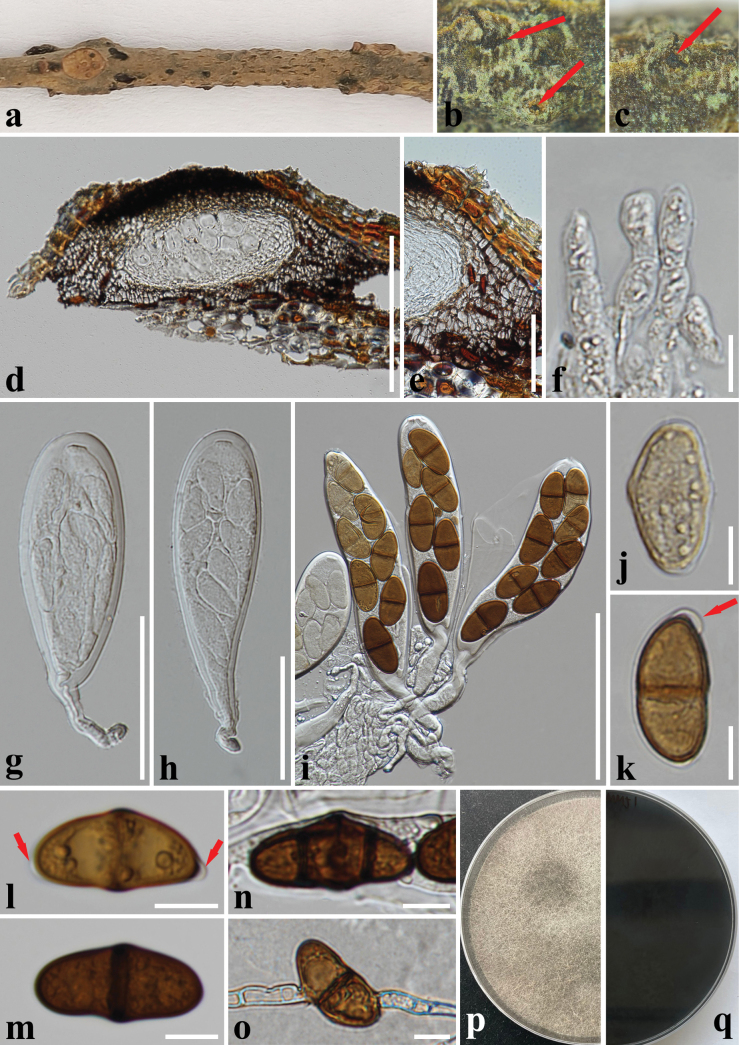
*Dothiorellamacadamiae* (CMUB 40066, holotype) **a–c** appearance of ascomata on the host surface **d** vertical section of an ascoma **e** peridium **f** hamathecium **g–i** asci **j–n** ascospores (arrows indicate mucilaginous polar appendages) **o** a germinating ascospore **p, q** colony on PDA (p-front and q-reverse views). Scale bars: 200 µm (**d**); 100 µm (**i**); 50 µm (**e, g, h**); 10 µm (**f, j–o**).

**Figure 4.**
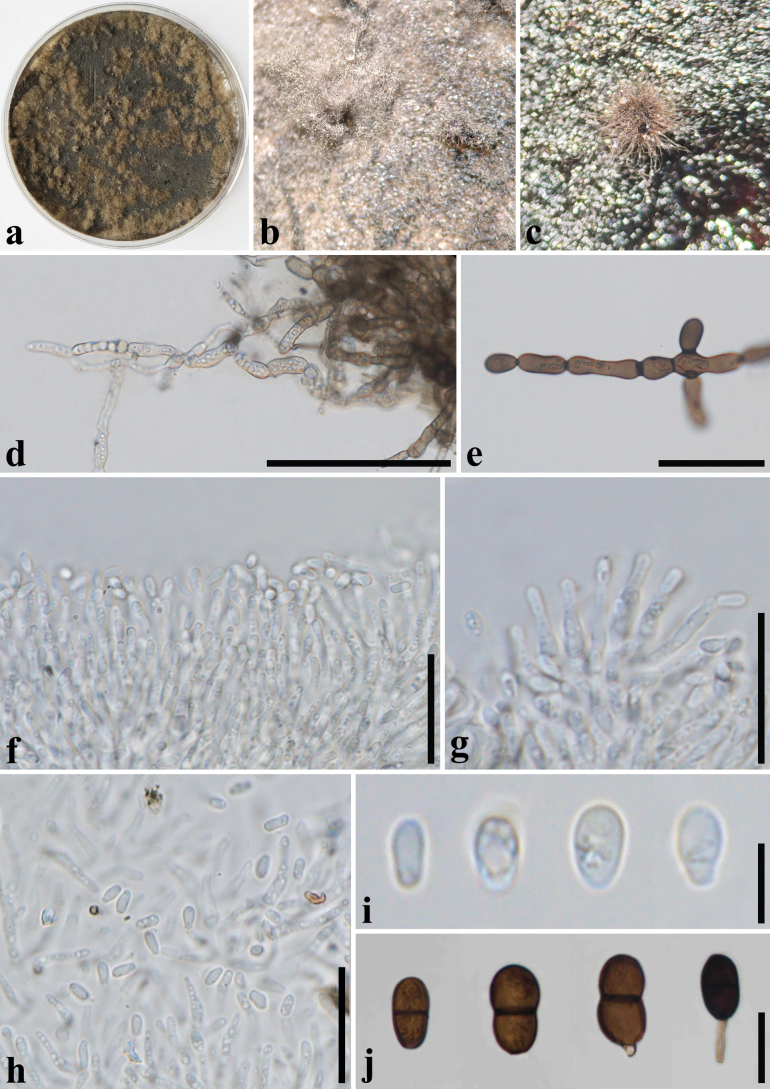
*Dothiorellamacadamiae* (SDBR-CMU512, ex-type) **a** colony on PDA**b, c** sporulating colonies on PDA**d, e** chlamydospores **f–h** conidiogenous cells with conidia **i–j** Conidia. Scale bars: 100 µm (**d**); 50 µm (**e**); 20 µm (**f–h, j**); 5 µm (**i**).

**Table 2.** Nucleotide comparisons of *Dothiorellamacadamiae* (SDBR-CMU512) with *D.albiziae* (MFLUCC 22-0057) and *D.thailandica* (MFLUCC 11-0438) based on ITS, *tef*1-α, and *TUB*2.

Taxa	ITS	*te*f1-α	*TUB*2
*D.albiziae* (MFLUCC 22-0057)	5/471 bp (1.0%, without gaps)	25/244 bp (10.2%, 8 gaps)	3/340 bp (0.8%, without gaps)
*D.thailandica* (CBS 133991)	8/519 bp (1.5%, without gaps)	30/285 bp (10.5%, 7 gaps)	6/371 bp (1.6%, without gaps)

#### ﻿ Phaeoacremonium

Taxon classificationFungiDiaporthalesTogniniaceae

W. Gams, Crous & M.J. Wingf.

3C5C585E-B575-544D-95B8-2D3E98AF015D

##### Notes.

*Phaeoacremonium* (Togniniaceae Réblová, L. Mostert, W. Gams & Crous) was introduced by Crous et al. (1996) with *P.parasiticum* (Ajello, Georg & C.J.K. Wang) W. Gams, Crous & M.J. Wingf. as the type species. In this genus, the asexual morph was recorded as *Phaeoacremonium*, and the sexual morph as *Togninia* Berl., which was introduced by Berlese (1900), with the type species *T.minima* (Tul. & C. Tul.) Berl. (Calabon et al. 2021; Phukhamsakda et al. 2022; Senanayake et al. 2023). Gramaje et al. (2015) synonymized *Togninia* under *Phaeoacremonium*, as it includes the majority of species, widely used by mycologists, and some *Togninia* species already have corresponding names in *Phaeoacremonium* (Calabon et al. 2021, 2024; Senanayake et al. 2023). The sexual morph of *Phaeoacremonium* is characterized by black ascomata with 8-spored asci, cylindrical, arising in acropetal succession, cylindrical-ellipsoidal to allantoid, hyaline, aseptate ascospores, and the asexual morph has hyaline to pigmented conidiophores, hyaline, cylindrical-ellipsoidal to allantoid conidia (Shang et al. 2021; Phukhamsakda et al. 2022; Senanayake et al. 2023; Calabon et al. 2024). In this study, we introduce a new species, *P.chiangmaiense*, from macadamia in Thailand. In addition, this is the first report of a *Phaeoacremonium* species from *Macadamia* species.

#### ﻿ Phaeoacremonium chiangmaiense

Taxon classificationFungiDiaporthalesTogniniaceae

X. Zhang & N. Suwannar. sp. nov.

0F259A37-CB2E-5BEC-923D-EFA468A7C75E

Index Fungorum: IF903217

Facesoffungi Number: FoF17085

Fig. 6

##### Etymology.

“*chiangmaiense*” refers to the location “Chiang Mai,” from where the holotype was collected.

##### Holotype.

CMUB 40065.

##### Description.

***Saprobic*** on dead twigs of *Macadamia* sp. **Sexual morph: *Ascomata*** 80–165 × 115–170 µm (x̄ = 136 × 146 µm, *n* = 20), immersed, solitary, globose to subglobose, dark brown to black, glabrous, ostiole with a long neck, neck straight or flexuous. ***Perithecial necks*** 75–160 µm high × 15–35 µm diam. (x̄ = 119 × 25 µm, *n* = 20), cylindrical, periphysate, ostiolar canals sulcate. ***Peridium*** 18–40 µm wide (x̄ = 29.4 µm, *n* = 25), comprising two section layers, the inner section layer composed of hyaline cells of ***textura prismatica***, the outer section layer comprising brown to dark brown cells of ***textura prismatica***. ***Hamathecium*** 3.5–5.5 µm wide (x̄ = 4.6 μm, *n* = 50), comprising cylindrical, hyaline, septate paraphyses, slightly inflated between the septa near their base and slightly contracted at the septa, longer than the asci. ***Asci*** 17–27 × 4–6 µm (x̄ = 20 × 5 µm, *n* = 50), 8-spored, arising in acropetal succession, unitunicate, apedicellate, cylindrical to clavate, apically rounded to truncate. ***Ascogenous hyphae*** hyaline, smooth-walled, septate, simple, 4–5 µm (x̄ = 4.7 μm, *n* = 10) at the base. ***Ascospores*** 4–7 × 1.2–2.5 µm (x̄ = 5.5 × 2 µm, *n* = 35), overlapping, hyaline, oblong to allantoid, aseptate, smooth-walled, rounded, and small guttules at both ends. **Asexual morph**: Undetermined.

##### Culture characteristics.

Ascospores germinating on PDA within 24 h at 28 °C, colony on PDA reaching 3 cm diam. after two weeks, culture from above flat, smooth surface, entire edges, white-yellow, low convex at the middle, forming tufts on the surface, wrinkled, reverse white to light reddish-brown from the edge to the center, wrinkled.

##### Material examined.

Thailand • Chiang Mai Province, 18°52'43"N, 99°13'15"E, 384 m elevation, on a dead branch of *Macadamia* sp., 24 November 2023, Xian Zhang, TCMM19, CMUB 40065 holotype; ex-type living culture, SDBR-CMU510, other living culture SDBR-CMU511.

##### GenBank number.

SDBR-CMU510 = ITS: PQ699720, *TUB*2: PQ736689, *ACT*: PQ736691, *tef*1-α: PQ724483, LSU: PQ699722; SDBR-CMU511 = ITS: PQ699721, *TUB*2: PQ736690, *ACT*: PQ736692, *tef*1-α: PQ724484, LSU: PQ699723.

##### Notes.

**Figure 5.**
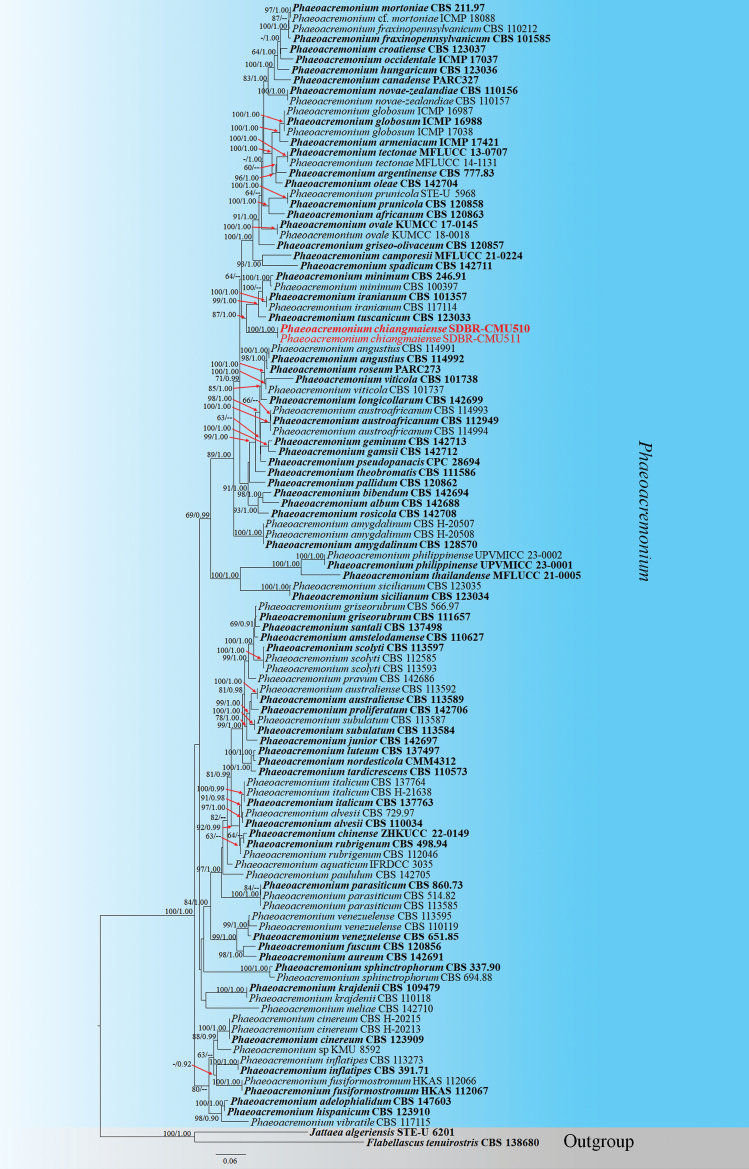
RAxML tree based on a combined dataset of ITS+*TUB*2+*ACT+tef*1-α+LSU gene sequences data, which comprised 2709 base pairs (ITS = 1–600 bp, *TUB*2 = 601–1237 bp, *ACT* = 1238–1499 bp, *tef*1-α = 1500–1816 bp, LSU = 1817–2709 bp). The best-scoring RAxML tree with a final ML optimization likelihood value of -29286.426085 is presented. The matrix had 1400 distinct alignment patterns, with 45.73% of undetermined characters or gaps. Estimated base frequencies were as follows: A = 0.224680, C = 0.290688, G = 0.252826, T = 0.231806; substitution rates: AC = 1.466922, AG = 3.673841, AT = 1.453901, CG = 1.188614, CT = 5.325402, GT = 1.000000; proportion of invariable sites I = 0.385193; gamma distribution shape parameter *α* = 0.691947. The bootstrap support values for ML are equal to or greater than 60%, and BI analysis values are equal to or greater than 0.90 PP at each node. The tree is rooted with *Flabellascustenuirostris* (CBS 138680) and *Jattaeaalgeriensis* (STE-U 6201). Newly generated species are shown in red, while the ex-type species are shown in bold.

The phylogenetic analyses showed that our isolates of *Phaeoacremoniumchiangmaiense* formed an independent lineage that is basal to three species of *Phaeoacremonium* (*P.iranianum* (CBS 101357, CBS 117114), *P.minimum* (CBS 246.91, CBS 100397), and *P.tuscanum* (CBS 123033)) with 87% ML and 1.00 PP support (Fig. 5). *Phaeoacremoniumchiangmaiense* (SDBR-CMU510, ex-type) was compared in ITS, *TUB*2, *ACT*, *tef*1-α, and LSU loci with *P.iranianum* (CBS 101357), *P.minimum* (CBS 246.91), and *P.tuscanum* (CBS 123033) based on nucleotides. The comparison results show that the *TUB*2, *tef*1-α, and *ACT* gene regions exhibit more than 10% differences (Table 3). Based on morphology, *P.iranianum*, *P.minimum*, and *P.tuscanum* were only recorded from asexual morphs, while *P.chiangmaiense* is recorded from the sexual morph; therefore, comparing these four species morphologically was not possible. Only 13 species of *Phaeoacremonium* were recorded from sexual morph (Hausner et al. 1992; Mostert et al. 2006; Hu et al. 2012; Huang et al. 2018; Calabon et al. 2021; Phukhamsakda et al. 2022). *Phaeoacremoniumchiangmaiense* is similar to other *Phaeoacremonium* species by having black ascomata, with asci arising in acropetal succession, hyaline ascogenous hyphae, and allantoid, reniform ascospores (Gramaje et al. 2015; Calabon et al. 2021; Phukhamsakda et al. 2022; Senanayake et al. 2023). *Phaeoacremoniumchiangmaiense* can be distinguished from other *Phaeoacremonium* species by its lack of branched hamathecium, overlapping and oblong ascospores (Gramaje et al. 2015; Phukhamsakda et al. 2022; Senanayake et al. 2023). Thus, we introduce *P.chiangmaiense* as a new species based on morphology phylogenetic analysis results.

**Figure 6.**
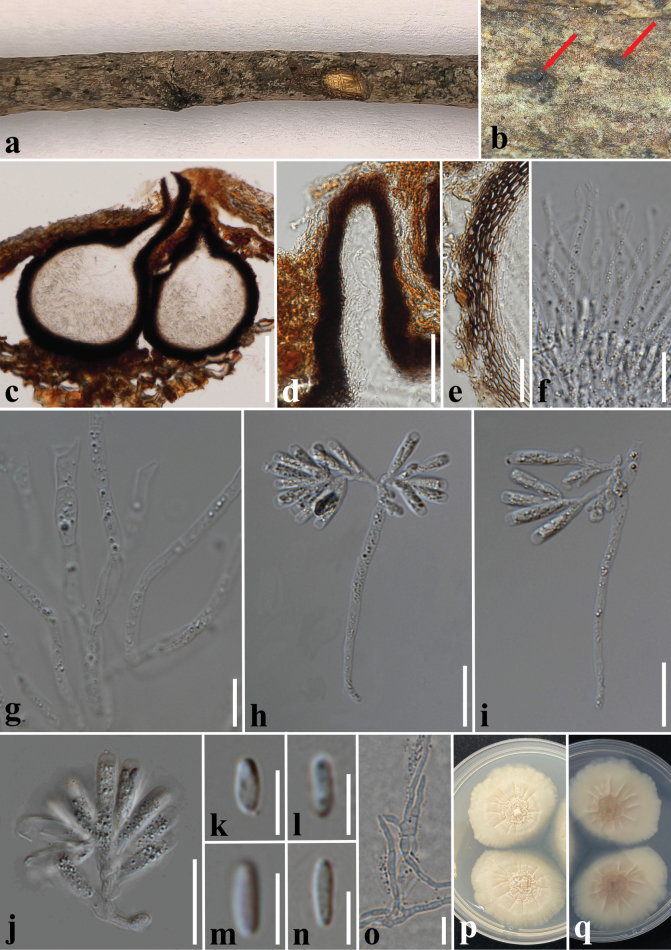
*Phaeoacremoniumchiangmaiense* (CMUB 40065, holotype) **a, b** appearance of ascomata on the host surface **c** vertical section of ascomata **d** ostiolar canal **e** section of peridium **f, g** hamathecium **h–j** ascogenous hyphae with asci attached **k–n** ascospores **o** germinated ascospores **p, q** colonies on PDA (p-front and q-reverse views). Scale bars: 100 µm (**c**); 40 µm (**d**); 20 µm (**e, f, h–j**); 10 µm (**g, o**); 5 µm (**k–n**).

**Table 3.** Nucleotide comparisons of *P.chiangmaiense* (SDBR-CMU510) with *P.iranianum* (CBS 101357), *P.minimum* (CBS 246.91), and *P.tuscanum* (CBS 123033), based on ITS, *TUB*2, *ACT*, and *tef*1-α; all of them were compared excluding gaps.

Taxa	ITS	*TUB*2	*ACT*	*tef*1-α
*P.iranianum* (CBS 101357)	20/544 bp (3.7%)	60/528 bp (11.3%)	37/260 bp (14.2%)	43/264 bp (16.3%)
*P.minimum* (CBS 246.91)	22/522 bp (4.2%)	78/498 bp (15.7%)	42/260 bp (16.2%)	42/267 bp (15.7%)
*P.tuscanum* (CBS 123033)	22/585 bp (3.8%)	70/596 bp (11.7%)	40/237 bp (16.9%)	38/269 bp (14.1%)

#### ﻿ Melomastia

Taxon classificationFungiBotryosphaerialesBotryosphaeriaceae

Nitschke ex Sacc.

E2CAB138-444E-5CE7-84A7-E21A45F9BCE0

##### Notes.

*Melomastia* (Pleurotremataceae Walt. Watson) was established by Saccardo (1875) to accommodate *M.mastoidea* (Fr.) J. Schröt (=*Melomastiafriesii* Nitschke) as the type species (Kang et al. 1999). *Melomastia* is characterized by immersed, ostiolar ascomata, brown to dark brown and comprising several layers of peridium, flexuose and filamentous paraphyses, 8-spored, cylindrical asci, ovoid, hyaline, 1–11-septate, fusiform to oblong ascospores with rounded or acute ends, with or without gelatinous sheath ascospores (Norphanphoun et al. 2017; Dayarathne et al. 2020; Li et al. 2022; Kularathnage et al. 2023; Xu et al. 2024). Norphanphoun et al. (2017) introduced *Melomastiaitalica* Norph., Camporesi, T.C. Wen & K.D. Hyde based on morphological characteristics and multi-locus phylogeny analyses, and the study revealed that *M.italica* and *Dyfrolomycesmaolanensis* Jin F. Zhang, Jian K. Liu, K.D. Hyde & Zuo Y. Liu form a distinct lineage, leading *D.maolanensis* to be synonymized under *M.maolanensis*. Additionally, *Dyfrolomyces* and *Melomastia* species exhibit morphological similarities; however, the relationship between these two genera remains unclear due to the limited availability of sequence data for *Melomastia* compared to the closely related genera and the change in ascomata morphology with different habitats. The generic delimitation within the family Pleurotremataceae has limited taxonomic significance when it is based on morphological characteristics; thus, 11 species of *Dyfrolomyces* were synonymized under *Melomastia* by Li et al. (2022) after they noted the lack of discernible morphological differences between the two genera. However, phylogenetic analyses revealed that *Melomastiatiomanensis* K.L. Pang, Alias, K.D. Hyde, Suetrong & E.B.G. Jones and *M.chromolaenae* (Mapook and K.D. Hyde) W.L. Li, Maharachch. & Jian K. Liu form a well-supported basal clade within the *Melomastia* lineage. Based on these findings and morphological characteristics, Kularathnage et al. (2023) reinstated *Dyfrolomyces* to accommodate *M.tiomanensis* and *M.chromolaenae*, and these two species can be distinguished from other *Melomastia* species by having spindle-shaped, 6–11-septate ascospores (Pang et al. 2013; Phukhamsakda et al. 2020; Kularathnage et al. 2023). Currently, *Melomastia* contains 66 epithets in Index Fungorum (http://www.indexfungorum.org/Names/Names.asp, accessed on 30 September 2024). However, the type species *M.mastoidea* still lacks the available sequence data (Li et al. 2022; Kularathnage et al. 2023).

#### ﻿ Melomastia puerensis

Taxon classificationFungiBotryosphaerialesBotryosphaeriaceae

R.F. Xu & Tibpromma, MycoKeys 103: 75 (2024)

94D66386-37F8-5FBC-A297-EA7D71410B91

Index Fungorum: IF901419

Facesoffungi Number: FoF15195

Fig. 8

##### Holotype.

ZHKU 23-0106.

##### Description.

***Saprobic*** on dead twigs of *Macadamiaintegrifolia*. **Sexual morph: *Ascomata*** 300–650 × 430–605 µm (x̄ = 521 × 516 µm, *n* = 20), visible as black raised dots on the host surface, solitary, semi-immersed, dark brown to black, subglobose or irregular pyriform, carbonaceous, papillate. ***Ostiolar*** central, carbonaceous, brown to dark brown to black. ***Peridium*** 30–80 µm wide (x̄ = 52 µm, *n* = 25), comprising two section layers: inner section layers hyaline to brown cells of ***textura prismatica***, outer section layer, brown to black cells of ***textura prismatica***. ***Hamathecium*** 2–4 µm wide (x̄ = 3 μm, *n* = 60), hyaline, filiform, septate, branched, pseudoparaphyses, longer than asci. ***Asci*** 166–235 × 5.6–9.9 µm (x̄ = 196 × 8.1 µm, *n* = 30), 8-spored, bitunicate, cylindrical, flexuous, smooth-walled, apically obtuse, with an ocular chamber, short-pedicellate. ***Ascospores*** 19–30 × 5–8 µm (x̄ = 26 × 6.5 µm, *n* = 50), hyaline, fusiform, uniseriate, 3-septate, narrow towards the apex and obtuse or conical ends, constricted at the septa, smooth-walled, without mucilaginous sheath or appendages, with guttules in each cell. **Asexual morph**: Undetermined.

##### Material examined.

China • Yunnan Province, Baoshan City, 24°48'18"N, 99°22'36"E, 1199.6 m elevation, on a dead branch of *Macadamiaintegrifolia*, 30 July 2022, Xian Zhang, MBC85, GMB1173, GMB1174, new host record.

##### GenBank number.

GMB1173 = LSU: PQ669573, SSU: PQ669629, *tef*1-α: PQ685655; GMB1174 = LSU: PQ669574, SSU: PQ669630, *tef*1-α: PQ685656.

##### Known host and distribution.

On a dead branch of *Heveabrasiliensis* in China (Xu et al. 2024), on a dead branch of *Macadamiaintegrifolia* in China (this study).

##### Notes.

**Figure 7.**
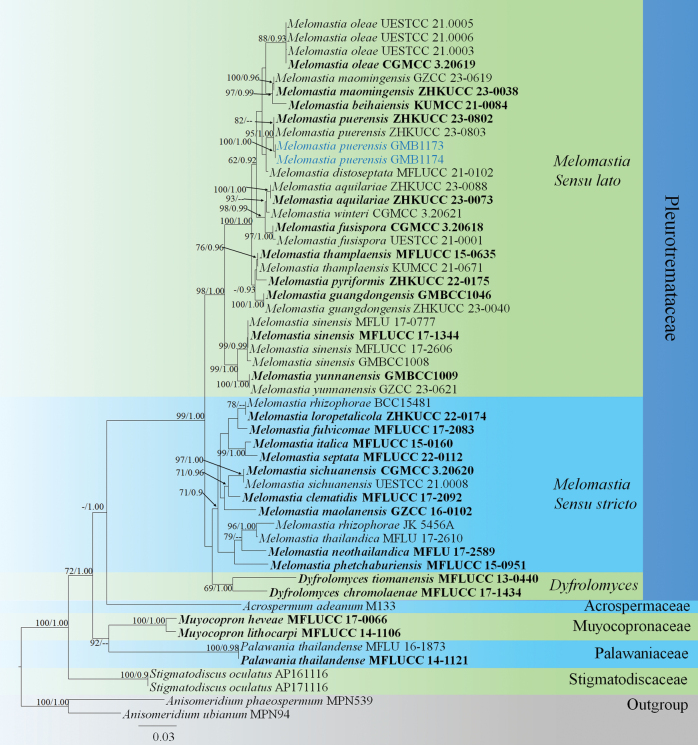
RAxML tree based on a combined dataset of LSU+SSU+*tef*1-α gene sequences. The topology of the trees generated by both maximum likelihood (ML) and Bayesian inference (BI) analyses exhibited high similarity. The RAxML tree with a final ML optimization likelihood value of -12726.117171. The aligned matrix had 933 distinct alignment patterns, with 22.57% of undetermined characters or gaps. Parameters for the GTR+I+G model of the combined LSU, *tef*1-α, and the SYM +I+G model of the combined SSU were as follows: estimated base frequencies A = 0.239385, C = 0.263871, G = 0.289634, T = 0.207110; substitution rates AC = 0.851550, AG = 2.071762, AT = 1.124966, CG = 0.971448, CT = 7.978925, GT = 1.000000; the proportion of invariable sites I = 0.478880; and gamma distribution shape parameter *α* = 0.661129. Bootstrap support values for ML equal to or greater than 60% and PP equal to or greater than 0.90 are given above the nodes. New records are in blue, while the ex-type species are in bold.

*Macadamiapuerensis* was reported by Xu et al. (2024), who isolated it from *Heveabrasiliensis* (Willd. ex A.Juss.) Müll.Arg., in Pu’er City, Yunnan Province, China. In the phylogenetic analyses, our strain *M.puerensis* (GMB1173) clustered with *M.puerensis* (ZHKUCC 23–0802, ex-type) with 100% ML and 1.00 PP support (Fig. 7). The nucleotide comparisons showed that our strain is not significantly different from ZHKUCC 23–0802 in LSU (0/842 bp (0%), without gaps), SSU (3/1035 bp (0.3%), without gaps), and *tef*1-α (0/903 bp (0%), without gaps). Morphologically, our collection is nearly identical to the holotype (ZHKU 23-0106), including the ascospores size (19–30 × 5–8 µm vs. 20–30 × 5–8 μm). Thus, we identified our strain as *M.puerensis*, representing a new host record on *M.integrifolia*. Additionally, this marks the first report of *Melomastia* associated with *Macadamia* (Table 4).

**Figure 8.**
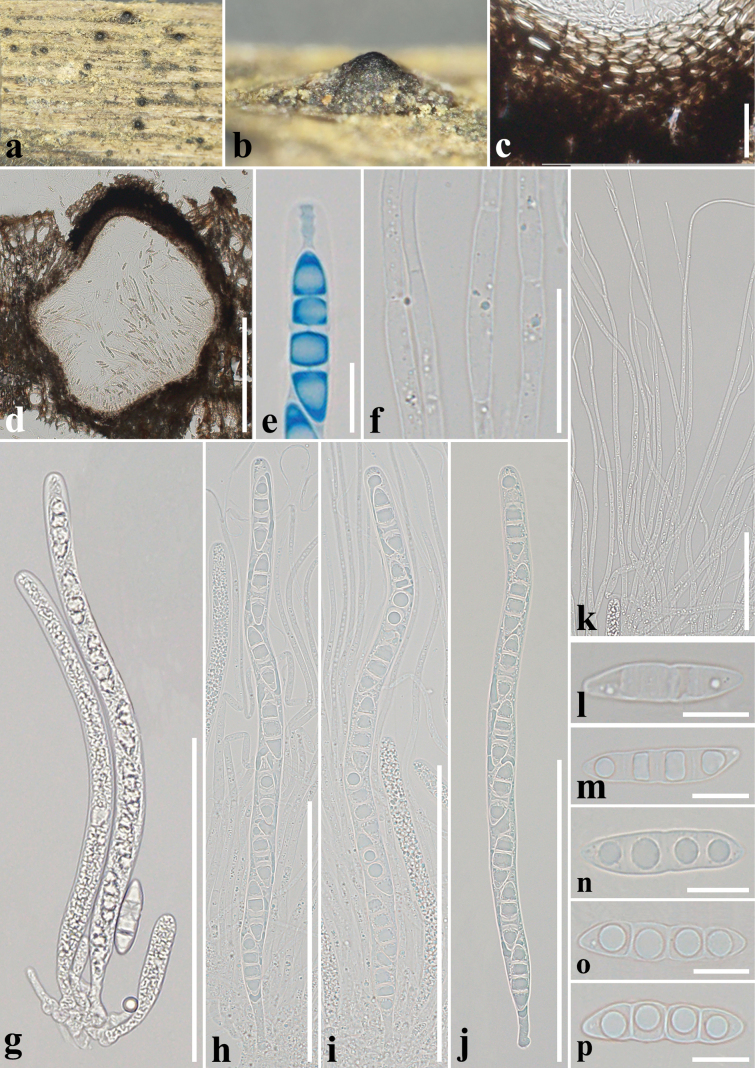
*Melomastiapuerensis* (GMB1173, new host record) **a, b** appearance of ascomata on the host surface **c** section of peridium **d** vertical section of an ascoma **e** ocular chamber in lactophenol cotton blue reagent **f, k** hamathecium **g–j** asci **l–p** ascospores. Scale bars: 200 µm (**d**); 100 µm (**g–j**); 50 µm (**k**); 20 µm (**c, f**); 10 µm (**l–p**).

**Table 4.** Occurrence of known hosts of *Melomastia* species and their distribution.

*Melomastia* species	Host records	Location	References
*M.antarctica*	*Pernettyamucronata*	Argentina	Spegazzini (1887)
*M.aquatica*	Unknown	China	Hyde (1992)
*M.aquilegiae*	*Aquilegiakarelini*	Kirghiz SSR	Index Fungorum (2024)
*M.calami*	*Calamus* sp.	Philippine	Ciferri (1928)
*M.beihaiensis*	*Chromolaenaodorata*	China	Senanayake et al. (2023)
*M.calligoni*	*Calligonum* sp.	Central Asia	Koshkelova and Frolov (1973)
*M.carinata*	*Ephedra*	Iran	Bateson et al. (1922)
*M.chilensis*	*Sophoramacrocarpa*	Chile	Petrak (1921)
*M.chromolaenae*	*Chromolaenaodorata*	Thailand	Mapook et al. (2020)
*M.clematidis*	*Clematissikkimensis*	Thailand	Phukhamsakda et al. (2020)
*M.clypeata*	*Salixmartiana*	Brazil	Sydow (1923)
*M.coffeae*	*Coffearobusta*	Central African Republic	Saccas (1981)
*M.constricta*	*Malusturkmenorum*	Turkmen SSR	Frolov (1967)
	*Cydoniaoblonga*	Central Asia	Koshkelova and Frolov (1973)
*M.corylina*	*Corylus* sp.	Luxemburg	Feltgen (1901)
*M.distoseptata*	Undetermined dead branch	India	Hongsanan et al. (2020)
*M.fulvicomae*	*Clematisfulvicoma*	Thailand	Phukhamsakda et al. (2020)
*M.fusispora*	*Oleaeuropaea*	China	Li et al. (2022)
*M.graminicola*	*Sorghumvulgare*	French	Saccas (1954b)
*M.haloxyli*	*Haloxylonaphyllum*	Kazakh SSR	Index Fungorum (2024)
*M.heteroderma*	Unknown	Cuba	Sydow (1936)
*M.heveae*	*Heveabrasiliensis*	Africa	Saccas (1954a)
*M.hyalostoma*	*Colavera*	Ivory Coast	Luc (1951)
*M.italica*	*Vitisvinifera*	Italy	Norphanphoun et al. (2017)
*M.jaapiana*	Betulaceae	Germany	Hein and Gerhardt (1981)
*M.kazachstanica*	*Ammodendronconollyi Haloxylonpersicum*	Central Asia	Koshkelova and Frolov (1973)
*M.lignicola*	*Betulapendula*	Germany	Harms et al. (1910)
*M.loropetalicola*	*Loropetalumchinense*	China	Dong et al. (2023)
*M.mastoidea*	*Chaenomelesspeciosa*	Ukraine	Schröter (1894a)
	*Cornussanguinea*	Denmark	Munk (1957)
	*Deutziacorymbosa*	India	Mueller (1958)
	*Fraxinusexcelsior*	Poland	Mulenko et al. (2008)
	*Fraxinus* sp.	Denmark	Munk (1957)
	*Lantanainvolucrata*	Bermuda	Vizioli (1923)
	*Lonicerapericlymenum*	Denmark	Munk (1957)
	*Loniceraquinquelocularis*	India	Bose and Mueller (1967)
	*Loniceraxylosteum*	Denmark	Munk (1957)
		Poland	Mulenko et al. (2008)
		Russia	Popov et al. (2008)
	*Osmanthusfragrans*	Ukraine	Dudka et al. (2004)
	*Populustremula*	Denmark	Munk (1957)
	*Rubiaperegrina*	Portugal	de Sousa Dias and Lucas (1972)
	*Sambucusnigra*	Denmark	Munk (1957)
	*Symphoricarpos* sp.	Denmark	Munk (1957)
	*Syringa* sp.	Denmark	Munk (1957)
		England	Dennis (1978)
	*Viburnumopulus*	Denmark	Munk (1957)
		Poland	Mulenko et al. (2008)
	*Metasphaeriamacounii*	Canada, British, Columbia	Schröter (1894b)
*M.metasequoiae*	*Metasequoiaglyptostroboides*	Ukraine	Dudka et al. (2004)
*M.mangrovei*	*Rhizophora* sp.	Thailand	Hyde et al. (2013)
*M.maolanensis*	Undetermined dead branch	China	Zhang et al. (2017)
*M.marinospora*	*Kandeliacandel*	Brunei	Hyde et al. (2013)
*M.neothailandica*	*Rhizophora* sp.	Thailand	Dayarathne et al. (2020)
*M.nigrificans*	Salicis	Luxemburg	Saccardo et al. (1882)
*M.oleae*	*Oleaeuropaea*	China	Li et al. (2022)
*M.pallidispora*	*Trematosphaeriapallidispora*	Italy	Norphanphoun et al. (2017)
*M.phetchaburiensis*	*Rhizophoraapiculata*	Thailand	Hyde et al. (2017)
*M.popuschoji*	*Amygdalusturcomanica*	Turkmen SSR	Frolov (1967)
*M.prorumpens* = *Trematosphaeriaprorumpens = Zignoëllaprorumpens*	Pine	Germany	Saccardo (1883)
*M.puerensis*	*Heveabrasiliensis*	China	Xu et al. (2024)
*M.pyriformis*	Undetermined dead branch	China	Kularathnage et al. (2023)
*M.rhizophorae*	*Rhizophoraapiculata*	Thailand	Hyde (1992)
*M.salicicola* = *Zignoëllasalicicola*	*Salixalba*	Vaucluse Galliae	Fabre (1883)
*M.saxauli*	*Haloxylonpersicum*, *Salsolaarbuscula*, *Salsolarigida*		Koshkelova and Frolov (1973)
*M.sedi*	*Sedumacre*	Crimean SSR	Gucevic (1967)
*M.septata*	Undetermined dead branch	Thailand	Hyde et al. (2023)
*M.sichuanensis*	*Oleaeuropaea*	China	Li et al. (2022)
*M.sinensis*	*Camelliasinensis*	Thailand	Hyde et al. (2018)
*M.shastensis*	Abiesmagnificavar.shastensis	California	Earle (1904)
*M.thailandica*	*Marinacvicennia*	Thailand	Hyde et al. (2016)
*M.thamplaensis*	Undetermined dead branch	Thailand	Zhang et al. (2017)
*M.tiomanensis*	*Rhizophora* sp.	Malaysia	Pang et al. (2013)
*M.winteri*	*Oleaeuropaea*	China	Li et al. (2022)
*M.yezoensis*	*Sasakurilensis*	Japan	Hino (1961)

## ﻿Discussion

This study introduces two new species and one new record of microfungi isolated from macadamia based on morphological and phylogenetic analyses. Botryosphaeriaceae contains 22 genera (Wijayawardene et al. 2022). Only seven species have been reported as macadamia-associated fungi in Botryosphaeriaceae, viz., six pathogenic fungi: *Botryosphaeriaribis* (= *Neofusicoccumribis*), *Lasiodiplodiairaniensis*, *L.theobromae*, *L.pseudotheobroma*, *Neofusicoccumaustrale*, and *N.parvum*, and one lifestyle unidentified species (*Diplodia* sp.) (Akinsanmi et al. 2015; Akinsanmi and Searle 2016; Jeff-Ego and Akinsanmi 2019; Tan et al. 2019; Farr and Rossman 2024). In this study, we introduce one new species in *Dothiorella* (*D.macadamiae*), from Chiang Mai Province, Thailand, and this is the first report of macadamia-associated saprobic fungi in *Dothiorella*. In this genus, some species have been reported from their sexual morphs, and some are asexual morphs, so we cannot distinguish them well from morphological characteristics; therefore, combining the ITS, *tef*1-α, and *TUB*2 genes is necessary to identify the species relationships. In addition, the type species of *D.pyrenophora* lacks multi-gene sequence data; thus, the type species needs fresh collection, pure cultures, and sequence data (Wu et al. 2024). Our analyses show that the *tef*1-α gene sequence of our isolations has more than a 10% bp difference compared with other genes (Table 2); therefore, we suggest that the *tef*1-α gene is important to reveal the phylogenetic placement of *Dothiorella*, which is also mentioned in Phillips et al. (2013) and Yang et al. (2017).

Li et al. (2022) synonymized *Dyfrolomyces* under *Melomastia*, but Kularathnage et al. (2023) reinstated *Dyfrolomyces* based on morphological characteristics and phylogenetic analyses, suggesting that earlier conclusions may require reassessment. This ongoing research keeps us intrigued about future findings. Meanwhile, the classification of these two genera has remained ambiguous due to their similar morphological characteristics and the lack of comprehensive molecular data.

Togniniaceae contains one genus (*Phaeoacremonium*), and Pleurotremataceae contains three genera (*Dyfrolomyces*, *Melomastia*, and *Pleurotrema*) (Wijayawardene et al. 2022; Index﻿ Fungorum 2025), but no species have been reported from macadamia. In this study, we introduce one new species in *Phaeoacremonium*, viz. *P.chiangmaiense*, and one new host record, viz. *M.puerensis*, as macadamia-associated fungi. The two new species and а new record found on macadamia increase the host distribution and geographical distribution of species in this family, enriching the fungal diversity of macadamia. This research has the potential to significantly impact our understanding of macadamia-associated fungi and the broader fungal diversity, highlighting the need for more saprobic fungi from a broader geographical area associated with macadamia based on multi-gene phylogenetic analyses that are necessary.

## Supplementary Material

XML Treatment for Dothiorella

XML Treatment for Dothiorella macadamiae

XML Treatment for Phaeoacremonium

XML Treatment for Phaeoacremonium chiangmaiense

XML Treatment for Melomastia

XML Treatment for Melomastia puerensis

## ﻿Additional information

### Author ORCIDs

Xian Zhang https://orcid.org/0000-0001-6097-8922

Saowaluck Tibpromma https://orcid.org/0000-0002-4706-6547

Samantha C. Karunarathna https://orcid.org/0000-0001-7080-0781

Tian-Ye Du https://orcid.org/0000-0003-2105-1803

Li-Su Han https://orcid.org/0000-0001-5380-9928

Abdallah M. Elgorban https://orcid.org/0000-0003-3664-7853

Jaturong Kumla https://orcid.org/0000-0002-3673-6541

Chanokned Senwanna https://orcid.org/0000-0002-1008-4514

Dong-Qin Dai https://orcid.org/0000-0001-8935-8807

Nakarin Suwannarach https://orcid.org/0000-0002-2653-1913

Hao-Han Wang https://orcid.org/0000-0002-2128-7894

### Data availability

All of the data that support the findings of this study are available in the main text.
